# *Pichia kudriavzevii* KKU20 and *Saccharomyces cerevisiae* Exhibit Comparable Performance in Rice Straw Ensiling for Feed Efficiency and Growth of Thai Native Beef Cattle

**DOI:** 10.1155/vmi/9966809

**Published:** 2026-04-27

**Authors:** Anusorn Cherdthong, Natdanai Kanakai, Pachara Srichompoo, Metha Wanapat, Suphakon Pramotchit, Chanon Suntara

**Affiliations:** ^1^ Tropical Feed Resources Research and Development Center (TROFREC), Department of Animal Science, Faculty of Agriculture, Khon Kaen University, Khon Kaen, 40002, Thailand, kku.ac.th

**Keywords:** ensiled rice straw, *Pichia kudriavzevii* KKU20, *Saccharomyces cerevisiae*, Thai native beef cattle

## Abstract

**Objective:**

Objective: The study aimed to assess the effect of two yeast strains, *Pichia kudriavzevii* KKU20 and *Saccharomyces cerevisiae*, on the ensiling process of rice straw (RS) and its impact on nutritional digestibility, ruminal fermentation, energy utilization, and growth performance of Thai native bulls.

**Methods:**

Eight Thai native bulls were randomly assigned to two groups: One group was fed RS ensiled with *Pichia kudriavzevii* KKU20 (*n* = 4), and the other group was fed RS ensiled with *Saccharomyces cerevisiae* (*n* = 4). The experiment included an 18‐day adaptation period followed by a 60‐day feeding period. The bulls received a concentrate diet formulated to meet their protein requirements.

**Results:**

No significant differences (*p* > 0.05) were observed between the groups in terms of daily concentrate intake, roughage intake, overall intake, average daily gain (ADG), and feed conversion ratio (FCR). Similarly, ruminal parameters showed no significant differences between the two yeast treatments, including ruminal pH, ammonia concentration, blood urea nitrogen levels, total volatile fatty acids (TVFAs), and protozoal populations. Energy efficiency and nitrogen efficiency were also unaffected by the yeast strain used (*p* > 0.05).

**Conclusion:**

Although no significant differences were found, *Pichia kudriavzevii* KKU20, a rumen‐derived yeast strain, demonstrated comparable potential to *Saccharomyces cerevisiae*, a widely used commercial yeast with known efficacy. This suggests that *P. kudriavzevii* KKU20 could be a promising alternative for ruminant nutrition.

## 1. Introduction

Rice straw (RS) is a major agricultural byproduct in Thailand, with annual production ranging from 20 to 32 million tons. Despite its potential, RS is often regarded as a waste [1]. Traditionally, farmers have resorted to burning unused RS, which has significantly contributed to global pollution levels [2]. However, in recent years, using RS as roughage feed for ruminants has emerged as a more sustainable solution, enhancing the inherent value of this agricultural byproduct while reducing its environmental impact [3]. Although RS has potential as ruminant roughage, its low digestibility remains a challenge due to its high silica and lignin (LN) content and low concentrations of vitamins and minerals. The lignification of supporting tissues and the presence of lignocellulose and hemicellulose (HC) are the primary factors limiting digestibility [4]. Multiple approaches have been employed to improve the nutritional composition and utilization of RS, including physical, chemical, and biological methods [5]. Physical methods involve cutting and chopping RS into smaller pieces, typically 2–3 mm in length. However, these methods are labor‐intensive and difficult to implement at the farm scale [6]. Chemical treatments, such as using acidic or alkaline solutions, have been explored to optimize RS’s nutritional content and break down its fiber structure [7]. However, these treatments are rarely used in practice because they may pose risks to cattle’s health [8]. In contrast, biological methods, which are the most widely used, are considered safer and more environmentally friendly [9].

Among biological methods, the use of yeast has been shown to have a noteworthy impact on improving RS digestibility and rumen fermentation [10]. Yeast strains are promising because of their enzymatic activity, ability to modulate rumen microbial populations, and potential to enhance fiber degradation. A particularly effective strategy is the application of yeast during the ensiling process, rather than supplementing it directly into cattle diets. This approach is effective because the ensiling environment allows yeast to preferment fibrous substrates, thereby reducing the complexity of structural carbohydrates such as neutral detergent fiber (NDF) and crude fiber (CF) while increasing the content of soluble nutrients [11–13]. Specifically, the use of yeast species such as *S. cerevisiae*, *Pichia kudriavzevii* KKU20, and *Candida tropicalis* KKU20 has been shown to increase crude protein (CP), enhance the production of volatile fatty acids (VFAs), a primary energy source for ruminants, and improve overall digestibility [11, 13, 14]. Furthermore, this bioprocessing technique can improve the aerobic stability of silage and reduce contamination from mycotoxins [12]. When compared with untreated RS, yeast‐treated roughages generally provide higher CP levels, improved digestibility, and greater energy availability, which demonstrates the rationale for applying yeast during ensiling instead of directly supplementing it to cattle. However, a previous study [11] compared RS treated with different yeast species but did not include untreated RS as a control, limiting the ability to evaluate the true improvement of yeast‐treated straw over untreated straw. The resulting increase in nutritional value is demonstrated *in vivo*, as previous experiments have demonstrated that certain yeast strains, particularly those isolated from the rumen of dairy cows, can significantly increase milk protein content when used to ferment RS *in vivo* trials [14]. However, most previous studies have focused on dairy cows, while their application in beef cattle remains limited. The nutritional requirements of beef cattle differ from those of dairy cows, particularly during various production phases. For instance, lactating dairy cows require feed with a higher nutritional quality than that of growing beef cattle. These differences in feed requirements likely influence the rumen environment, potentially resulting in varying effects on feed efficiency and performance. This variability highlights a critical research gap: the impact of rumen‐isolated yeasts on beef cattle is still largely unexplored.

While the metabolic advantages of Crabtree‐negative yeasts such as *Pichia kudriavzevii* for improving forage quality are well‐established, enhancing fiber degradation without producing excess ethanol [11, 14], most research has focused on *in vitro* digestibility or dairy production outcomes. However, comprehensive data on the *in vivo* effects of these advanced fermented forages on the growth performance, nutrient utilization, and overall metabolic responses in beef cattle remain limited. This knowledge gap highlights the need to evaluate their practical application and physiological impact within beef production systems.

The present study addresses this gap by evaluating the effects of the rumen‐isolated yeast *P*. *kudriavzevii* KKU20 in Thai native beef cattle. The selection of *P. kudriavzevii* KKU20 is particularly noteworthy, as previous research has demonstrated its ability to proliferate effectively, produce high levels of cellulase, and interact uniquely with the rumen environment [10]. It is hypothesized that *P. kudriavzevii* KKU20 will improve feed intake, digestibility, ruminal fermentation, energy utilization, and growth performance in beef cattle to a degree comparable to that of the commercial yeast *Saccharomyces cerevisiae*, which has consistently shown favorable results in many studies. Therefore, the objective of this study was to evaluate the effects of these two yeast strains on the ensiling process of RS and to assess their impacts on nutritional digestibility, ruminal fermentation, energy utilization, and growth performance in Thai native beef cattle.

## 2. Materials and Methods

### 2.1. Ethical Procedure

The research conducted in this study obtained approval from the Animal Ethics Committee (Record No. IACUC‐KKU 49/67) based on the Institutional Guidelines of Khon Kaen University, National Research Council of Thailand. Throughout the study, animal welfare was ensured by minimizing stress during handling, providing appropriate housing and nutrition, and following humane procedures during all experimental activities, in compliance with ethical standards.

### 2.2. Yeast Obtained

Crabtree‐negative ruminal yeast, *Pichia kudriavzevii* KKU20 (strain CBS 5147T; MH545928), was obtained from a previous study [15]. This yeast was isolated, screened, and identified from the rumen of Thai–Holstein Friesian dairy cattle and then processed into a dry form. *P. kudriavzevii* was selected for this study due to its ability to enhance the quality of feedstuffs and support efficient metabolic activity under aerobic conditions.

The distinction between Crabtree‐negative and Crabtree‐positive yeasts is crucial in yeast selection. Crabtree‐negative yeasts, such as *P. kudriavzevii*, prioritize respiration over fermentation under aerobic conditions, producing biomass and carbon dioxide as the primary products. Conversely, Crabtree‐positive yeasts, such as *S*. *cerevisiae*, tend to produce ethanol under aerobic conditions by inhibiting pyruvate dehydrogenase, leading to reduced efficiency and lower cell growth.

The Crabtree‐positive yeast, *S. cerevisiae*, was obtained from a commercial source (Perfect Yeast Co., Ltd., Ubon Ratchathani, Thailand). This yeast is widely used in agricultural applications but is less efficient under aerobic conditions compared to Crabtree‐negative species.

To preserve viability and maintain quality, all yeasts were stored in sterile, vacuum‐sealed 20 mL glass vials. These vials were kept refrigerated at 4°C for short‐term storage, while long‐term preservation was achieved by maintaining the yeasts at −80°C in an ultra‐low freezer. These storage methods ensured the stability and functionality of the yeast strains throughout the experimental period.

### 2.3. Preparation of Rice Straw Ensiled With Yeast

The experimental work was conducted at the Tropical Feed Resources Research and Development Center (TROFREC), Khon Kaen, Thailand, using RS collected between March and April 2022. Yeasts were categorized into two dietary groups, Crabtree‐positive and Crabtree‐negative. In a previous study [15], *P*. *kudriavzevii* KKU20 exhibited superior biomass production, cellulase activity, and cell counts (∼10^6^ cells/mL). In comparison, our study employed a commercial *S*. *cerevisiae* strain obtained from Perfect Yeast Co. Ltd, Ubon Ratchathani, Thailand.

### 2.4. Preparation of Yeast Medium Solutions

The yeast medium was prepared by mixing 250 g of molasses and 100 g of urea in 1000 mL of tap water, and the pH was adjusted to 3.5 using formic acid. Yeast propagation was carried out for 72 h at 39°C under continuous aeration, following a modified method [15]. For each propagation batch, approximately 10^8^ cfu was inoculated, resulting in a final cell concentration of 1.11 × 10^8^–1.41 × 10^8^ cells/mL. The fermented yeast medium was then applied to RS at a ratio of 2:1 (medium: RS, w/w), corresponding to an inoculation level of approximately 1 × 10^6^–10^7^ cells/g fresh RS. The moisture content of the straw was adjusted to 650–750 g/kg based on the recommendations of a previous report [16]. For ensiling, 10 kg of RS was vacuum‐sealed in plastic bags and stored under anaerobic conditions at room temperature for 14 days, following a previously described procedure [15].

### 2.5. Animals and Experimental Design

A group of eight Thai native bulls, aged approximately 18 months, with an average starting body weight (BW) of 286 ± 41.16 kg, were housed individually in 5 × 5 m stalls, each cleaned regularly to maintain animal health. The bulls had free access to mineral blocks (BOSLIC, PK Kaset Co., Ltd., Surin, Thailand) and clean water throughout the experimental period.

The bulls were assigned to two treatment groups using a complete randomized design (CRD) approach. The treatment groups consisted of *P. kudriavzevii* KKU20 and *S. cerevisiae* ensiled with RS. The experiment had an 18‐day adaptation period before the 60‐day feeding period to allow the bulls to gradually adjust to the experimental diets, avoid feed refusal of the yeast‐treated RS, and stabilize rumen fermentation before data collection. The Thai native cattle received the basic diet two times each day, with feedings occurring beginning at 7 a.m. and 4 p.m. During the study period, a concentrated feed containing a CP concentration of 15.50% was given to the animals daily, at a quantity ranging from 1.55% to 1.65% of the dry matter (DM) of the BW. This dietary treatment was formulated to achieve a daily weight gain of 0.50 kg, in accordance with the recommendations provided in a previous standard [17]. Afterward, Ad libitum access to ensiled RS was provided, as described in a previous study [18].

### 2.6. Dietary Nutrient Analysis

The diet samples were oven‐dried at 60°C for 48 h for sample preparation and ground to pass a 1‐mm screen. DM was determined by oven‐drying at 105°C for 24 h [19]. CP (ID 984.13), ether extract (EE), and ash were analyzed [19]. NDF and acid detergent fiber (ADF) were determined [20]. Acid detergent LN (ADL) was analyzed [21]. Gross energy (GE) was measured using a bomb calorimeter (AC 500, LECO, St. Joseph, MI, USA).

### 2.7. The Process for Collecting Samples and Chemical Analysis

During the feeding trial, we recorded the daily intake of roughages and concentrated diet for each animal by carefully weighing both the provided and leftover feed. Samples of the concentrated and ensiled RS were collected weekly and then separated into two equal portions. The first component was subjected to DM analysis, after which it was dried inside an oven at an average temperature of about 100°C, while the following portion was stored at a temperature of −20°C for further study. The first component passed through DM analysis. Following that, the composite samples were frozen and then further divided into smaller portions for the study of their nutritional composition and GE content.

The animals were individually housed in metabolic cages specifically designed to allow free movement and to facilitate the efficient and separate collection of urine and feces for sampling. A fraction of the overall fresh fecal samples, about 5 g/kg, was divided into two separate portions. The first portion of each daily fecal sample was used to evaluate the DM composition, while the next portion of the daily fecal samples was combined into an overall pooled sample. Following the collection process, the fecal samples, weighing 500 g each, were frozen at −20°C until analysis. The dietary materials were melted, followed by drying at 60°C for 72 h. Subsequently, each of the samples was put through a drying process before grinding using a Cyclotech Mill (Tecator, Hoganas, Sweden) fitted with a 1 mm screen to enhance the evaluation of their chemical composition. To determine the energy content of the urine, we formed cotton into a sharp, round shape and then placed it into preweighed calorimeter crucibles to measure their respective weights. The resulting weights of the crucibles, now containing the urine samples, were then measured.

On the last day of the feeding study, the bulls continued their daily routine. Blood samples were obtained and centrifuged at 500 g for an average of 10 min to separate plasma. The obtained plasma was stored at 20°C for subsequent analysis of blood urea nitrogen levels [22]. Rumen fluid samples (100 mL) were collected using a stomach tube attached to a vacuum pump at two time points: immediately before feeding and four hours after feeding. To minimize salivary contamination, the first three ruminal fluid draws were discarded. The standard stomach‐tube sampling method was used to collect rumen fluid [23]. Following that, we quickly measured the pH of the ruminal fluid using a pH indicator (HANNA Instruments HI 8424 microcomputer, Singapore). Following pH measurement, rumen fluid samples were divided into two portions. An aliquot of 45 mL was immediately acidified with 9 mL of 1 M H_2_SO_4_ in a 60 mL plastic container to preserve samples for subsequent analysis of ammonia nitrogen (NH_3_‐N) and VFAs (acetate, propionate, and butyrate). The ruminal fluid samples were centrifuged at a speed of 16,000 × *g* for 15 min to help evaluate NH_3_‐N and VFA. The concentration of NH_3_‐N was determined by distillation [19]. The concentration of VFAs was estimated using gas chromatography [24]. This included using a DB‐Wax column manufactured by Agilent Technologies, USA, with dimensions of 30 m in length, 0.25 mm in diameter, and a 0.25 μm film thickness. Bacterial and protozoal populations were counted using a hemocytometer (Boeco, Hamburg, Germany) under a light microscope at 400x magnification. Rumen fluid samples were diluted with 10% formalin at a ratio of 1:9 (rumen fluid: formalin) before counting.

### 2.8. Calculations

The calculation of energy utilization was performed as follows:

(1) GEI, Mcal/day=DM  intake  of  dietkg/day×GE  content  in  dietMcal/kg  DM,

where GEI = GE intake and GE = GE.

(2) FE, Mcal/day=fecal  DMkg/day×fecal  GEMcal/kg  DM,

where FE = fecal energy; UE, Mcal/day = urine yield (kg/day) × GE of urine (Mcal/kg); UE = urine energy; DEI = digestible energy (DE) intake; GEI = GEI; and DEI and Mcal/day = GEI (Mcal/day) − FE (Mcal/day).

(3) Metabolizable  energy  intake MEI, Mcal/day=DEI−UE+methane energy.

Methane energy, Mcal/day = (8 × GEI) ÷ 100.

Total digestible nutrients (TDNs) were calculated as follows:

(4) TDN%=% digestible crude protein+% digestible crude fiber+% digestible N−free extract2.25×% digestible ether extract.

The determination of HC and cellulose (CL) content was performed using the following approach:

(5) HC %=%NDF−%ADF,CL %=%ADF−%ADL.

### 2.9. Statistical Analysis

In a completely randomized design, the statistical study was performed using the analysis of variance (ANOVA) device within Statistical Analysis Systems (SAS) 2002. The model used was represented as Yij = *µ* + τi + ɛij, where Yij represents the observed values for the treatments, μ represents the overall mean, τi signifies the treatment effect of ensiled RS, and ɛij denotes the random error. Data were presented as averages from eight Thai native bulls, along with standard errors of means. Significance was determined at *p* < 0.05 using the Duncan multiple ranking test.

## 3. Results

### 3.1. Chemical Analysis to Determine the Nutritional Content of the Diet

Table 1 summarizes the chemical composition of the concentrate and roughage sources used in this experiment. The RS ensiled with *P. kudriavzevii* KKU20 had a CP content of 5.22%, an EE of 0.82%, a NDF of 69.96%, an ADF of 42.40%, CL at 31.27%, HC at 27.56%, and LN at 11.14%. On the other hand, RS ensiled with *S. cerevisiae* demonstrated slightly higher CP at 6.63%, lower NDF at 68.72%, comparable ADF at 42.87%, CL at 31.38%, and lower HC and LN levels at 25.85% and 11.49%, respectively. These variations highlight *S. cerevisiae*’s potential to increase protein content while slightly reducing fiber fractions compared to *P. kudriavzevii* KKU20. In addition, both ensiled RSs showed substantial improvements in protein content and reductions in fiber fractions compared to untreated RS (2.54% CP, 80.6% NDF, and 53.1% ADF), indicating the beneficial effects of ensiling with yeasts.

**TABLE 1:** Chemical composition of nutrients in the beef cattle diet.

Ingredients	Concentrate	Rice straw	Rice straw ensiled with *P. kudriavzevii* KKU20	Rice straw ensiled with *S. cerevisiae*
Cassava chip (%)	47.75	—	—	—
Soybean meal (%)	14.00	—	—	—
Rice bran (%)	22.00	—	—	—
Palm kernel (%)	14.00	—	—	—
Salt (%)	1.00	—	—	—
Urea (%)	1.00	—	—	—
Premix (%)	0.25	—	—	—
Chemical composition				
Dry matter (DM) (%)	90.11	93.4	33.36	33.04

**% of dry matter**				

Organic matter	94.14	88.8	86.36	87.68
Crude protein	15.50	2.54	5.22	6.63
Ether extract	4.71	0.80	0.82	0.72
Neutral detergent fiber	21.24	80.6	69.96	68.72
Acid detergent fiber	11.76	53.1	42.4	42.87
Cellulose	10.18	45.1	31.27	31.38
Hemicellulose	9.48	27.5	27.56	25.85
Lignin	1.59	8.00	11.14	11.49
Gross energy (Mcal/kgDM)	3.93	3.30	3.47	3.55

### 3.2. Feed Intake, Digestive Efficiency, and Livestock Performance

The effects of ensiled RS with different yeast species on feed intake, digestive efficiency, and livestock performance in Thai native beef cattle are illustrated in Table 2. Ensiling RS with either *P. kudriavzevii* KKU20 or *S. cerevisiae* exhibited no significant impact (*p* > 0.05) on daily concentrate intake (kg/day), roughage intake (kg/day), and overall intake (kg/day), with values ranging from 4.24 to 4.50, 3.52 to 3.73, and 7.76 to 8.23 kg/day, respectively. Overall nutrient digestibility and TDN were not affected by treatments. Furthermore, the performance of beef cattle fed RS ensiled with different yeast species was not significantly affected (*p* > 0.05). Average daily gain (ADG) remained similar between treatments, while feed conversion ratio (FCR) values were 12.52 and 13.7 for *P. kudriavzevii* KKU20 and *S. cerevisiae*, respectively. Although this difference was not statistically significant, the numerically lower FCR in the KKU20 group suggests a trend toward improved feed efficiency, which could be of practical relevance in large‐scale production systems.

**TABLE 2:** Effect of rice straw ensiled with different yeast species on animal performance.

Item	*P. kudriavzevii* KKU20	*S. cerevisiae*	SEM	*p* value
Dry matter intake				
Concentrate intake kg/day	4.24	4.5	0.35	0.07
% BW	1.55	1.65	0.05	0.69
g/kg BW^0.75^	67.8	71.92	2.79	0.51
Roughage intake kg/day	3.52	3.73	0.25	0.73
% BW	1.29	1.39	0.09	0.47
g/kg BW^0.75^	52.94	56.07	3.38	0.31
Total intake kg/day	7.76	8.23	0.55	0.36
% BW	2.94	2.95	0.08	0.79
g/kg BW^0.75^	118.35	120.27	2.16	0.28
ADG (kg/day)	0.72	0.68	0.16	0.94
FCR	12.52	13.7	2.68	0.98

**Nutrient digestibility (%)**				

Dry matter	79.33	84.01	3.71	0.26
Organic matter	50.33	41.62	2.18	0.59
Ether extract	91.16	93.06	1.67	0.17
Crude protein	86.83	89.89	2.32	0.15
Neutral detergent fiber	74.59	78.59	4.41	0.35
Acid detergent fiber	83.87	87.05	2.92	0.27
Hemicellulose	96.24	97.78	0.61	0.15
Cellulose	72.9	78.91	2.59	0.82
Total digestible nutrient (%)	75.41	79.31	0.23	0.54

*Note:* BW^0.75^ = metabolic weight.

Abbreviations: ADG = average daily gain; FCR = feed conversion ratio; SEM = standard error of the mean.

### 3.3. Ruminal Fermentation and Blood Metabolites

The results showed no statistically significant differences (*p* > 0.05) between bulls offered *P. kudriavzevii* KKU20 and those given *S. cerevisiae* (Table 3). Ruminal pH ranged from 7.09 to 7.25, which is slightly above but still close to the optimal physiological range (6.0–7.0) for ruminal fermentation. NH_3_‐N (3.36–4.79 mg/dL) fell within the normal range of 2–5 mg/dL, indicating adequate nitrogen availability for microbial protein synthesis. Blood urea nitrogen (9.00–11.50 mg/dL) was at the lower end of the expected physiological range (10–20 mg/dL) but remained within acceptable limits. Total VFAs (59.24–86.31 mM) were slightly lower than the commonly reported optimal range (80–150 mM), though still adequate to support microbial fermentation.

**TABLE 3:** Evaluating the impact of ensiled rice straw on ruminal pH, ammonia concentrations, blood urea nitrogen levels, total volatile fatty acids (TVFAs), and protozoal populations in Thai native bulls.

Item	*P. kudriavzevii* KKU20	*S. cerevisiae*	SEM	*p* value
Ruminal pH				
0 h	7.22	7.25	0.08	0.82
4 h	7.10	7.09	0.06	0.91
Mean	7.16	7.17	0.08	0.97
Ruminal ammonia nitrogen (mg/dL)				
0 h	4.51	4.79	0.52	0.66
4 h	3.98	3.36	0.5	0.60
Mean	4.28	3.83	0.9	0.80
Blood urea nitrogen (mg/dL)				
0 h	9.50	9.00	2.07	0.42
4 h	11.50	10.67	0.65	0.09
Mean	10.50	9.71	1.63	0.50
Bacteria number (× 10^8^ cell/mL)				
0 h	9.27	8.49	0.27	0.42
4 h	9.93	9.80	0.70	0.44
Mean	9.60	9.14	0.56	0.33
Protozoa number (× 10^7^ cell/mL)				
0 h	1.00	1.30	0.14	0.25
4 h	1.13	0.80	0.15	0.62
Mean	1.06	1.09	0.16	0.08
Total volatile fatty acids (mM)				
0 h	66.63	59.24	5.36	0.49
4 h	86.31	71.59	8.8	0.79
Mean	73.19	65.41	7.5	0.69
Acetic acid (mole/100 mol)				
0 h	76.40	76.42	0.63	0.96
4 h	77.49	76.77	0.4	0.92
Mean	76.94	76.59	0.54	0.8
Propionic acid (mole/100 mol)				
0 h	13.98	14.02	0.86	0.79
4 h	12.52	13.41	0.35	1.00
Mean	13.25	13.71	0.68	0.51
Butyric acid (mole/100 mol)				
0 h	9.63	9.56	0.49	0.98
4 h	9.99	9.83	0.44	0.09
Mean	9.81	9.69	0.44	0.36

Abbreviation: SEM = standard error of the mean.

### 3.4. Energy Utilization and Efficiency

The evaluation of the energy utilization and efficiency in Thai native bulls is outlined in Table 4. Notably, various energy intake and utilization parameters were examined, including GEI (Mcal/day), FE, UE, methane energy (CH4 energy, Mcal/day), DEI, and MEI. In addition, ratios such as DEI/GEI, MEI/GEI, and MEI/DEI were evaluated. Statistical analysis revealed no significant differences (*p* > 0.05) among treatments. Specifically, GEI ranged from 29.65 to 31.80 Mcal/day, DEI from 23.03 to 23.69 Mcal/day, and MEI from 14.52 to 14.58 Mcal/day. The corresponding ratios (DEI/GEI = 0.71–0.72, MEI/GEI = 0.43–0.44, and MEI/DEI = 0.60–0.61) were also nearly identical between groups. This numerical similarity across energy intake and utilization parameters supports the conclusion that both yeast treatments provided comparable energy efficiency and, consequently, comparable animal performance.

**TABLE 4:** Comparison of the responses of energy utilization and efficiency of the Thai native bull.

Item	*P. kudriavzevii* KKU20	*S. cerevisiae*	SEM	*p* value
Gross energy intake				
Mcal/day	29.65	31.8	2.2	0.24
Mcal/kg BW^0.75^	0.45	0.49	0.02	0.8
Fecal energy				
Mcal/day	9.25	8.78	0.56	0.5
% Gross energy intake	31.41	28.62	3.23	0.33
Mcal/kg BW^0.75^	0.14	0.14	0.01	0.3
Urine energy				
Mcal/day	6.54	5.9	0.76	0.09
% Gross energy intake	20.23	19.16	3.14	0.51
Mcal/kg BW^0.75^	0.1	0.09	0.01	0.46
Methane energy				
Mcal/day	14.52	14.58	2.94	0.6
Mcal/kg BW^0.75^	0.22	0.22	0.04	0.93
Digestible energy intake				
Mcal/day	23.69	23.03	1.87	0.25
Mcal/kg BW^0.75^	0.36	0.35	0.03	0.74
% Gross energy intake	71.73	71.38	3.16	0.26
Metabolizable energy intake				
Mcal/day	14.52	14.58	2.94	0.6
% Gross energy intake	43.5	44.23	5.89	0.86
Mcal/kg BW^0.75^	0.22	0.22	0.04	0.93
Energy efficiency				
DEI/GEI	0.72	0.71	0.03	0.26
MEI/GEI	0.43	0.44	0.06	0.86
MEI/DEI	0.6	0.61	0.06	0.78

Abbreviations: DEI = digestible energy intake; GEI = gross energy intake; MEI = metabolizable energy intake; SEM = standard error of the mean.

## 4. Discussion

This research used several yeast species to evaluate the CP content of ensiled RS, which was found to be 5.22% and 6.63%. However, nonensiled RS typically has a CP content of 3%–4% [25]. Crabtree yeast was reported to improve CP quality by 5.89%–7.12% and enhance fermentation [15]. Fermented straw with Crabtree yeast contained NDF levels of 62.5%–71.5%, ADF levels of 39.1%–45.2%, and ADL levels of 6.1%–6.5%, which were lower compared to the fiber content in untreated RS. Therefore, isolated yeast can produce cellulase enzymes and break down fiber [25]. Crabtree yeast has been reported to produce cellulase at 0.004–0.08 units/mL, which may support fiber degradation in the rumen [26]. It has been confirmed that the use of fermented yeast in straw can reduce the amount of fiber it contains [27].

In Thailand, RS typically offers the main source of roughage in the diet of beef cattle [28]. The research revealed a broad range of RS intake, ranging from 1.1% to 2.5% BW [29]. Furthermore, the intake of RS fermentation with various additions, such as molasses or enzymes, is approximately 1.3%–2.0% BW [24]. Consequently, the research revealed no significant difference in RS intake among bull cattle. The use of Crabtree yeast in ensiled RS was reported to have no significant effect on feed intake [15]. Furthermore, the intake of ensiled RS by dairy cows was estimated at approximately 1.0% of their BW. Moreover, the present study found that neither Crabtree yeast strain influenced beef cattle nutrient digestibility or TDNs. Similarly, the fermentation of RS with *Lactobacillus casei* TH14, molasses, and cellulase enzymes did not affect total intake in cattle across treatments [30]. However, our experiment was the fermentation of RS and yeast. Fermentation of RS with both strains of Crabtree yeast was reported to have no significant effect on total intake or TDN [15]. In contrast, the use of the yeast strain *P. kudriavzevii* KKU20 in dairy cows led to a notable 6.43% improvement in DM digestibility [31]. The lack of effect observed in beef cattle may be related to an insufficient inoculation rate or suboptimal fermentation quality of the ensiled RS, which limited improvements in fiber degradability [32–34].

The pH values ranged from 7.09 to 7.25 in this study. These values correspond to the optimal pH range of 6.2–7.2 for microbial activity [35]. Maintaining pH within this range supports microbial fermentation and promotes overall rumen function. The average ruminal NH_3_‐N levels in bulls in the current study varied from 3.36 to 4.79 mg/dL, which were lower than the range of 17.7–19.4 mg/dL in the previous report [15]. Moreover, when RS was treated with urea, NH_3_‐N concentrations increased from less than 2 to 9 mg/dL [36]. These variations can be partly explained by differences in dietary nitrogen content between dairy and beef cattle. In particular, beef cattle diets often contain lower CP concentrations (*N* values ranging from 1.87% to 2.45%) compared to the higher levels in lactating dairy cow diets (2.14%–2.88%) [37]. This reduced dietary N intake and the correspondingly lower nitrogen requirement in beef cattle may directly contribute to lower ruminal NH_3_‐N concentrations [38]. Physiologically, ruminal NH_3_‐N reflects the balance between dietary protein degradation and microbial nitrogen assimilation; values below 5 mg/dL, as observed in our study, may indicate limited nitrogen availability for optimal microbial growth [39, 40].

Furthermore, the BUN levels in this study (9.0–11.50 mg/dL) were not affected by treatment but were lower than the 13.3–17.30 mg/dL reported in lactating dairy cows fed ensiled RS with Crabtree yeast [15]. Since BUN is positively correlated with dietary nitrogen intake and ruminal NH_3_‐N concentrations [41], the BUN values observed in this study are consistent with previous findings in beef cattle fed low‐protein diets [42–44]. Physiologically, a reduction in dietary nitrogen intake not only leads to a direct decrease in circulating BUN but also limits the recycling of nitrogen back into the rumen, thereby influencing overall nitrogen metabolism. Specifically, it has been demonstrated that cattle on low‐protein regimens have smaller pools and lower net synthesis rates of BUN and ruminal NH_3_‐N. Consequently, the extent of urea recycling to the rumen diminishes as dietary protein decreases, which alters the overall nitrogen balance and metabolism of the animal [43, 45].

Feeding Thai native beef cattle ensiled RS with *P. kudriavzevii* KKU20 and *S. cerevisiae* had no effect on energy intake and did not negatively affect energy utilization, allowing animals to receive sufficient energy for growth and maintenance. Our results showed that GEI ranged from 0.45 to 0.49 Mcal/kg BW^0.75^/d, which is comparable to previously reported values of 0.25–3.06 Mcal/kg BW^0.75^/d in Thai native cattle [46]. In addition, the energy efficiency observed in the present study (DE‐to‐GE [DE/GE] = 0.71–0.72) is consistent with values reported for RS‐based feed additives [47]. The DE/GE ratio observed in this study, which was above 0.70, indicates that the dietary energy was well digested and metabolically available. This high level of energy utilization is particularly crucial for Thai native cattle, which are often managed on low‐quality forages such as fermented RS [47, 48]. Research has consistently shown that improving such forages through microbial fermentation enhances nutrient and energy digestibility, leading to a higher DE/GE ratio and consequently better growth performance and energy efficiency [47, 48]. Furthermore, a high DE/GE ratio is directly associated with increased energy absorption and reduced energy losses via feces and methane [47–49]. This implies that the ensiled RS supplemented with yeast, as used in our study, can provide a reliable source of utilizable energy, potentially reducing the need for expensive concentrate supplementation. Furthermore, a previous report demonstrated that using cassava pulp achieved an energy efficiency of 0.64–0.71 [50], suggesting that ensiled RS may be comparable or superior in energy utilization, thereby reinforcing its potential as a sustainable feed resource for beef cattle production systems.

## 5. Conclusions

Based on this study, it can be concluded that ensiled RS with *P. kudriavzevii* KKU20 and *S. cerevisiae* in the diet did not elicit any observable adverse effects on key parameters, including feed intake, ruminal fermentation, and energy and nitrogen utilization. The ruminal yeast strain *P. kudriavzevii* KKU20 exhibits fermentation potential comparable to that of the commercial yeast *S. cerevisiae* for ensiled RS. Future research should involve an extended feeding trial with a larger sample size and longer duration to assess the effects of ensiled RS with *P. kudriavzevii* KKU20 and *S. cerevisiae* on overall health, carcass characteristics, and meat quality.

## Author Contributions

Planning and design of the study: Anusorn Cherdthong, Natdanai Kanakai, Pachara Srichompoo, Metha Wanapat, Suphakon Pramotchit, and Chanon Suntara. Conducting and sampling: Anusorn Cherdthong, Natdanai Kanakai, Pachara Srichompoo, and Chanon Suntara. Statistical analysis: Anusorn Cherdthong, Natdanai Kanakai, Pachara Srichompoo, and Chanon Suntara. Manuscript drafting: Anusorn Cherdthong, Natdanai Kanakai, Pachara Srichompoo, Suphakon Pramotchit, and Chanon Suntara. Manuscript editing and finalizing: Anusorn Cherdthong, Natdanai Kanakai, Pachara Srichompoo, Metha Wanapat, and Suphakon Pramotchit.

## Funding

The authors express their sincerest gratitude to the Fundamental Fund of Khon Kaen University, which has received funding support from the National Science, Research, and Innovation Fund (NSRF). Research Program on the Research and Development of Winged Bean Root Utilization as Ruminant Feed, Increase Production Efficiency, and Meat Quality of Native Beef and Buffalo Research Group and Research and Graduate Studies, Khon Kaen University (KKU), were also acknowledged.

## Disclosure

All authors have read and agreed to the published version of the manuscript.

## Consent

The authors have nothing to report.

## Conflicts of Interest

The authors declare no conflicts of interest.

## Data Availability

The data that support the findings of this study are available from the corresponding author upon reasonable request.
